# Firing Without Wiring: How REM-like Sleep Reactivation Rebalances Excitation and Inhibition to Orthogonalize Memories

**DOI:** 10.64898/2026.10.11.757684

**Published:** 2026-10-21

**Authors:** Yoshimasa Kubo, Erik Delanois, Maxim Bazhenov

## Abstract

The role of Rapid Eye Movement (REM) sleep in memory consolidation remains poorly understood. Several influential ideas propose that REM sleep serves to eliminate crosstalk between memories, thereby orthogonalizing representations of similar ones; however, mechanistic confirmation remains sparse. Here we used recurrent neural networks trained with Equilibrium Propagation (EP), a biologically plausible training algorithm, to investigate the role of REM-like sleep in memory consolidation. We found that REM-like sleep reduces memory interference by decorrelating task-specific neuronal populations, isolating individual memory traces. During sleep, reactivation was dominated by the population encoding the most recent memory, which had taken over the inputs of older memories during new learning. The excitation/inhibition balance of inputs onto this population shifted toward inhibition, most strongly from older-memory populations, so that older memories again activated their own neurons. Spreading the same total synaptic change evenly across all target populations abolished this recovery. We call this mechanism reactivation-targeted pruning: synaptic weights onto the reactivated population decrease when its neurons fire while their presynaptic partners are silent. The mechanism held under a synapse-level Dale's law, and excitatory plasticity alone, with inhibitory synapses fixed, was sufficient. These findings suggest mechanistic principles underlying REM sleep's role in memory consolidation.

